# Safety and Efficacy of the Consumption of the Nutraceutical “Red Yeast Rice Extract” for the Reduction of Hypercholesterolemia in Humans: A Systematic Review and Meta-Analysis

**DOI:** 10.3390/nu16101453

**Published:** 2024-05-11

**Authors:** Efstratios Trogkanis, Maria A. Karalexi, Theodoros N. Sergentanis, Eleni Kornarou, Tonia Vassilakou

**Affiliations:** 1 Department of Public Health Policy, University of West Attica, 11521 Athens, Greece; s.trogkanis@sa.aegean.gr (E.T.); tsergentanis@uniwa.gr (T.N.S.); ekornarou@uniwa.gr (E.K.); 2 Department of Hygiene and Epidemiology, University of Ioannina School of Medicine, 45110 Ioannina, Greece; marykaralexi@windowslive.com

**Keywords:** red yeast rice extract, nutraceuticals, hypercholesterolemia, total cholesterol, LDL cholesterol, efficacy, safety, systematic review, meta-analysis

## Abstract

Previous studies have shown encouraging results regarding the efficacy and safety of nutraceuticals, such as “red yeast rice (RYR) extract”, on reducing hypercholesterolemia in humans. A systematic review and meta-analysis was conducted from January 2012 to May 2022. The search was strictly focused on clinical trials that examined the association between RYR extract consumption and parameters of the lipid profile in humans. Fourteen double-blinded clinical trials were identified. The interventions lasted 4–24 weeks. In most studies, there was one intervention group and one control group. RYR extract consumption statistically significantly reduced total cholesterol (mean absolute reduction: 37.43 mg/dL; 95% confidence interval [CI]: −47.08, −27.79) and low-density lipoprotein cholesterol (LDL-C; mean absolute reduction: 35.82 mg/dL; 95% CI: −43.36, −28.29), but not high-density lipoprotein cholesterol, triglycerides and apolipoproteins A-I and B. As regards the safety, RYR extract was considered a safe choice with neither threatening nor frequent side effects. The consumption of RYR extract by people with hypercholesterolemia was associated with statistically significant reduction in total cholesterol and LDL-C, whereas it was not associated with an increase in life-threatening side effects. Further research on specific subpopulations and outcomes could establish a consensus on determining the clinical benefits and potential risks, if any, of this nutraceutical.

## 1. Introduction

The Western lifestyle and dietary habits have been linked to a range of chronic diseases, including obesity, type 2 diabetes and cardiovascular disease [1]. Thus, policy makers and scientific and public health campaigns have focused on the adoption of healthy dietary measures aiming to prevent such diseases [2]. The principal target of these measures is the reduction in hypercholesterolemia, mainly focusing on the replacement of saturated fats with monounsaturated or polyunsaturated fats [3]. Several medications are currently used to reduce hypercholesterolemia, with statins being the most common and primarily used. Statins have been extensively studied in the past in terms of their multiple positive cardiovascular effects, as well as their potential adverse effects [4].

In recent years, newer categories of preparations have emerged, the “nutraceuticals”, which are widely available on the market following different regulations for their marketing approval compared to those of classic drugs [5,6,7,8]. However, there is lack of consensus regarding the safety and effectiveness of such nutraceuticals [9]. Since 2017, expert groups have started recommending the consumption of nutraceutical alternatives for the management of hypercholesterolemia in humans [10].

Among nutraceuticals, red yeast rice (RYR) extract, produced by the fungus *Monascus purpureus* during the fermentation of rice, is widely used to improve the lipidemic profile and reduce the risk of cardiovascular events [11,12]. The main component of this extract is monacolin K which is structurally identical to Lovastatin, a statin currently used to treat hypercholesterolemia in humans. Previous reviews and meta-analyses have examined the efficacy and safety of this nutraceutical, showing encouraging results [13,14,15,16]. However, most of the previously published meta-analyses included studies that were not double-blinded and of lower quality.

Current evidence is insufficient to establish consensus regarding the efficacy and safety of RYR extract supplementation in humans with hypercholesterolemia. To this end, we systematically searched the published literature up to the most recent publication, focusing strictly on clinical trials, to assess the effectiveness of RYR extract consumption on reducing parameters of the lipidemic profile in humans. We further examined in detail any potential adverse effects following the consumption of this nutraceutical to better inform the general population about the possible risks, if any.

## 2. Materials and Methods

### 2.1. Literature Search

The present systematic review and meta-analysis was conducted following the updated guidelines proposed by the Cochrane Collaboration [17], as well as the Preferred Reporting Items for Systematic Reviews and Meta-Analyses Statement [18] (Table S1). The present study is based on a Master of Science (MSc) thesis undertaken in the context of MSc in Public Health organized by the Department of Public Health Policy, University of West Attica, and as such, a protocol has been submitted to the MSc Programme beforehand (registration number: Uniwa 40535-20/05/2021).

We searched Medline database through PubMed, as well as Cochrane Central Register of Clinical Trials (CENTRAL), Excerpta Medica Database (Embase), Scopus, CINAHL and Clinicaltrials.gov, from January 2012 until May 2022 for original studies examining the safety and efficacy of the consumption of RYR extract for the reduction in hypercholesterolemia in humans, employing a predefined search algorithm (Appendix A).

The literature search was limited to studies published during the last 10 years, as well as to English publications.

### 2.2. Eligibility Criteria

Eligible articles included clinical trials that examined the use of RYR extract for the reduction in hypercholesterolemia in humans. When one or more eligible studies on the same research question were identified, the one with the largest number of component studies, usually the most recently published study, was included. Each identified study was cross-checked for its quality and issues of overlap before being included in the final set of eligible studies.

### 2.3. Data Extraction and Quality Assessment

Information was extracted on the name of the first author, year of publication, epidemiological design, sample size, interventions that were performed, and maximally adjusted effect size and 95% confidence intervals (CIs).

The data extraction database included study quality indicators based on the criteria of the National Heart, Lung, and Blood Institute (NHLBI) for the quality assessment of clinical trials [19]. Such criteria included a description of the study as randomized, treatment allocation concealed, participants and providers blinded to treatment group assignment, people assessing the outcomes blinded to the participants’ group assignments, groups similar at baseline in terms of important characteristics that could affect outcomes, ≤20% overall drop-out rate from the study at the endpoint, <15% differential drop-out rate at the endpoint, high adherence to the intervention protocols, other interventions avoided or similar in the groups, outcomes assessed using valid and reliable measures, sample size power ≥80%, outcomes reported or subgroups analyzed prespecified, and the use of intention-to-treat analysis. For each criterion, each study was assigned 1 point, with a maximum of 14 points. The eligible studies were considered of adequate quality if they were rated with more than 10 points.

### 2.4. Statistical Analysis

The primary outcome of this meta-analysis was the assessment of low-density lipoprotein cholesterol (LDL-C) levels, as well as the measurement of the proportion of change in LDL-C levels after the consumption of RYR extract. Secondary outcomes were the assessment of high-density lipoprotein cholesterol (HDL-C), total cholesterol (TC), triglycerides (TGs), apolipoprotein (Apo) A-I and Apo B levels, and the proportion of change in the levels of the above indicators following the consumption of RYR extract. In addition, the potential side effects after the consumption of RYR extract were also examined.

Statistical analyses included pooling of original studies. Random effects models were appropriately applied to calculate the pooled effect estimates, namely the Risk Ratios (RRs) and their 95% CIs for the categorical variables, as well as the Mean Difference (MD) and Standardized Mean Difference (SMD) for the linear variables. Between-study heterogeneity was assessed by using Q-test and I^2^ estimations (Higgins index); considerable heterogeneity was considered when I^2^ was >50% [17].

Statistical analyses were performed using Review Manager (Revman) version 5.3 (by Cochrane, London, UK).

## 3. Results

### 3.1. Description of Eligible Studies

**Figure 1:**
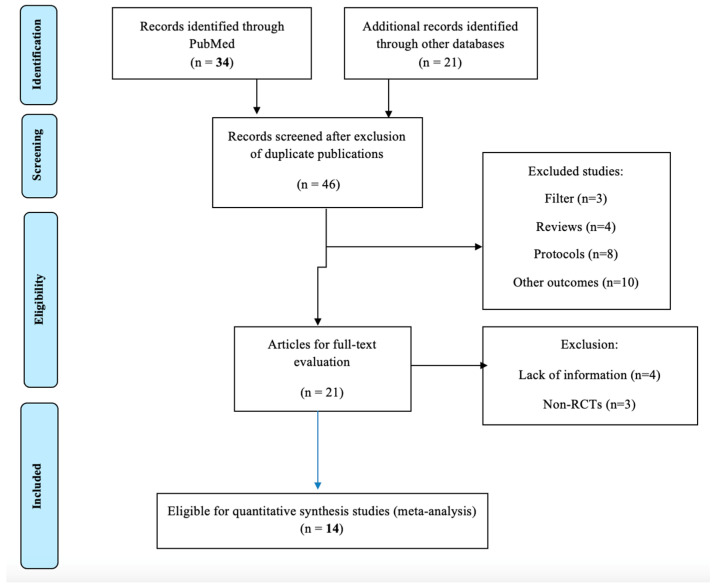
A flowchart of the successive steps in the systematic review process.

A flowchart of the successive steps in the systematic review process is shown in Figure 1. A total of 36 records were identified from PubMed using the search algorithm (Appendix A) and were assessed for eligibility. Twenty-one studies were additionally retrieved from other sources. Following the exclusion of duplicate publications, 46 studies were available for full-text evaluation. After the additional exclusion of 32 studies due to specific reasons, 14 studies were finally eligible to be included in the present systematic review and meta-analysis [20,21,22,23,24,25,26,27,28,29,30,31,32,33] (Figure 1). The quality assessment of the eligible studies is presented in Table S2. Five studies were rated with maximum points (*n* = 14), three studies were rated with 13 points, three with 12 points, two with 11 points, and one with 10 points.

The majority of studies were conducted in Europe (*n* = 10), whereas 3 studies were conducted in Asia and 1 in the USA (Table 1). The sample size of participants per study ranged from 18 to 142 persons, yielding a total of 705 persons with hypercholesterolemia who participated either in intervention or controls groups. The mean age of participants was 52.7 years (range: 43.8–61.4 years), while the average body mass index (BMI) was 26.1 kg/m^2^ (range: 23.4–30.3 kg/m^2^; Table 1).

The interventions lasted 4–24 weeks. In most studies (*n* = 11), there was one intervention group and one control group. Two studies used two intervention groups and one control group, whereas one study used only one intervention group. All nutraceutical compounds contained RYR in various concentrations and combinations. The RYR extracts were available either as tablets or sachets, whereas the total dose ranged from one to six tablets consumed one to three times per day. Table 2 presents the intervention characteristics of the included studies.

### 3.2. Efficacy of Red Yeast Rice Extract

#### 3.2.1. Effects on Total Cholesterol

**Figure 2:**
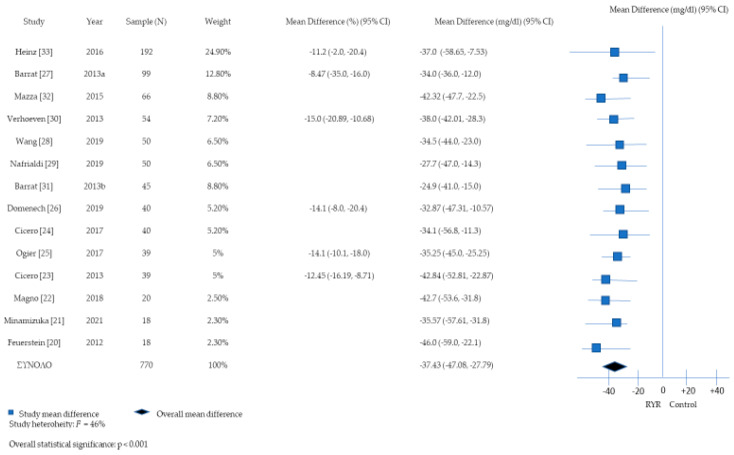
A forest plot describing the association between red yeast rice extract consumption and changes in total cholesterol (TC) levels [20,21,22,23,24,25,26,27,28,29,30,31,32,33].

Figure 2 depicts the forest plot of the meta-analysis (*n* = 14 study arms) on the effect of RYR extract consumption on TC levels. This meta-analysis showed a statistically significant summary mean absolute reduction in TC levels of 37.43 mg/dL (95% CI: −47.08, −27.79) in the intervention compared to the control group. Between-study heterogeneity was non-considerable (I^2^: 46%).

#### 3.2.2. Effects on Low-Density Lipoprotein Cholesterol

**Figure 3:**
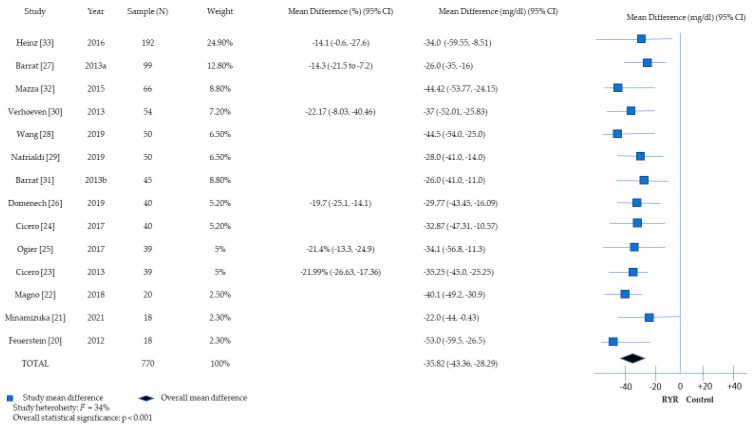
A forest plot describing the association between red yeast rice extract consumption and changes in low-density lipoprotein (LDL) cholesterol levels [20,21,22,23,24,25,26,27,28,29,30,31,32,33].

All studies reported a statistically significant reduction in the levels of LDL-C in the intervention group compared to the control group, as shown in Table 3. The meta-analysis (*n* = 14 study arms) showed a statistically significant summary mean absolute reduction of 35.82 mg/dL (95% CI: −43.36, −28.29) in LDL-C levels without considerable between-study heterogeneity (I^2^: 34%; Figure 3). In most studies, the reduction in LDL-C levels was assessed at the end of intervention (*n* = 4–16 weeks), whereas one study further reported that the reduction in LDL-C levels had been evident since the fourth week of intervention (proportion of change: −15.19%; 95% CI: 22.9, −7.5%) and thereafter plateaued up to the end of the study (*n* = 16 weeks) [31].

#### 3.2.3. Effects on High-Density Lipoprotein Cholesterol, Triglycerides and Apolipoproteins

Conducting a meta-analysis on other secondary outcomes, namely, the effects of RYR extract consumption on HDL-C, TG and apolipoprotein levels, was not feasible due to large between-study heterogeneity; thus, these effects are only described qualitatively.

The study by Heinz et al. (2016) showed a statistically significant reduction of 5.0% in the levels of TGs between week 1 and week 12 (*p* = 0.001) in the intervention group, but not in the control group [33]. The LDL-C/HDL-C ratio was also reduced by 14.1% in the intervention group, but this reduction was not statistically significant. Likewise, in the study by Ogier et al. (2013), the TG levels were significantly reduced by 12.2% after 16 weeks in the intervention compared to control group [25]. In the same context, the study by Mazza et al. (2015) reported a statistically significant mean reduction of 18.6 mg/dL in the levels of TGs (*p* < 0.001) [32].

In the study by Feuerstein and Bjerke (2012), non-statistically significant changes were noted in the levels of HDL-C and TGs at the end of the intervention, as well as in the levels of BMI and systolic and diastolic blood pressure [20]. The study by Cicero et al. (2013) also did not show statistically significant changes in the levels of TGs, HDL-C, apolipoproteins, glucose, creatinine and creatine phosphokinase (CPK) between the two study groups [23]. The second intervention study by Cicero et al. (2017) reported a statistically significant reduction in the levels of non-HDL-C by 31.2 mg/dL (95% CI: −45.5, −8.1; *p* < 0.001), as well as in the levels of high-sensitivity C-reactive protein (hs-CRP) by 0.4 mg/dL (95% CI: −0.9, −0.1; *p* < 0.019) [24]. There were non-significant changes in weight, BMI, blood pressure and heart rate in this study.

Positive results were also noted in the study by Barrat et al. (2013a) [31]. Notably, the levels of Apo-B, as well as the TC/HDL-C and LDL-C/HDL-C ratios, were significantly reduced at 4 weeks after the consumption of RYR extract, and were thereafter stabilized or slightly increased up to the 16th week of intervention. The second intervention study by Barrat et al. (2013b) showed non-significant changes in the levels of HDL-C and TGs after 4 weeks of consumption of RYR extract [27]. In the same context, the study by Magno et al. (2018) reported non-significant changes in the levels of HDL-C and TGs following 8 weeks of RYR extract consumption [22]. Similar non-significant results regarding the levels of TGs were also noted in the studies by Nafrialdi et al. (2019) [29] and Wang et al. (2019) [28].

The study by Domenech et al. (2019) showed statistically significant changes in the levels of non-HDL-C and Apo-B in the intervention group compared to the placebo group [26]. The reduction in Apo-B was −13.5% after 12 weeks of RYR extract consumption, whereas in the placebo group, the reduction in Apo-B levels was −2.9% (*p* < 0.001). Non-significant changes in the levels of TGs, HDL-C and Apo-A1 were also noted in that study. Lastly, the study by Minamizuka et al. (2021) showed a statistically significant mean reduction of 0.18 g/L (standard deviation: ±0.11 g/L, *p* = 0.011) in the levels of Apo-B after 8 weeks of RYR extract consumption [21].

### 3.3. Safety of Red Yeast Rice Extract

Eight of the fourteen studies reported data on the safety of RYR extract supporting the evidence that RYR extract consumption is considered safe, without having considerable side effects (Table 4). Furthermore, the reported side effects were rare and non-life-threatening. Particularly, two studies showed no side effects clearly related to RYR extract, given that all reported side effects were evident both in the intervention and the control group [21,29]. The most common side effects included gastrointestinal symptoms, such as constipation, flatulence, diarrhea (rarely), dyspepsia, nausea, bitter taste and abdominal pain (very rarely). Other symptoms included headache, eczema and cystitis. Very rare symptoms affecting the musculoskeletal system included myalgia, with concurrent increases in CPK levels, whereas the increase in the SGOT/SGPT ratio was rare. Of note is that the aforementioned side effects were not associated in a dose–response pattern with RYR extract consumption.

## 4. Discussion

### 4.1. Principal Findings

The present meta-analysis comprising data from 14 clinical trials found statistically significant effects of RYR extract consumption on the reduction in total and LDL cholesterol levels. The reduction in TC levels ranged from −11.2% to −19.2%, with a summary mean reduction of −37.43 mg/dL at the meta-analysis level. Likewise, the proportion of reduction in LDL-C levels ranged from −14.3% to −22.17%, yielding a summary mean reduction of −35.82 mg/dL. Regarding TG levels, four studies reported proportions of reduction ranging from −5.0% to −16.3%, whereas seven studies showed non-significant changes in the levels of TGs following the consumption of RYR extract. Lastly, a few studies found reductions in the levels of non-HDL-C (*n* = 3 studies), hs-CRP (*n* = 2 studies) and Apo-B (*n* = 1 study) in the intervention groups. With regard to the safety of RYR extract, the present systematic review provides evidence that the consumption of RYR extract may be considered a safe choice, with neither life-threatening nor frequent adverse events.

### 4.2. Interpretation of Findings

The present systematic review and meta-analysis found mean levels of reduction in LDL-C levels comparable to those of low-dose statins, which are more commonly used in the treatment of hypercholesterolemia [34]. Specifically, Pravastatin 10 mg, Simvastatin 10 mg, Fluvastatin 20 mg and Lovastatin 20 mg result in an average 20% reduction in LDL-C levels. Our results are also comparable to those of a previous meta-analysis which showed a mean reduction of 39.4 mg/dL in LDL-C levels after the consumption of monacolin K (mean dose = 10.8 mg per day) [35].

Congruently with the present study, previous systematic reviews and meta-analyses have also reported positive effects of RYR extract consumption [35,36], enhancing the evidence that the use of RYR extract seems to significantly reduce both total cholesterol and LDL-C to a similar extent. Overall, short-term clinical studies have shown that preparations with RYR extract have short-term positive effects in the treatment of hypercholesterolemia [10], while larger clinical trials have shown that the above nutraceutical may also contribute to a lower risk of cardiovascular events as secondary prevention in the long run [37].

The secondary prevention hypothesis is further supported by a recent meta-analysis [15] which aimed to examine the long-term effect of RYR extract consumption on the incidence of myocardial infarction. This study found that RYR extract consumption (dose: 1200 mg/day) was associated with a reduced risk of myocardial infarction (RR: 0.42; 95% CI: 0.34–0.52), revascularization (RR: 0.58; 95% CI: 0.48–0.71) and sudden death (RR: 0.71; 95% CI: 0.53–0.94). Moreover, RYR extract consumption reduced LDL-C levels by 20.70 mg/dL (95% CI: −24.51, −16.90), TC levels by 26.61 mg/dL (95% CI: −31.65, −21.58) and TG levels by 24.69 mg/dL (95% CI: −34.36, −15.03). In contrast, HDL-C levels were significantly increased by 2.71 mg/dL (95% CI: 1.24, 4.17). Therefore, based on these data, it seems that RYR extract may be associated with positive long-term cardiovascular effects.

However, information on the safety of RYR extract is lacking or is only partially measurable. Moreover, there is no standardized way of producing the specific food drug by the various companies and suppliers worldwide. Thus, in the USA, warnings were issued by the relevant food safety authorities regarding the stability of the production methods of RYR extract, since some components of RYR extract that are hepatotoxic may be produced during its maturation [38]. A recent systematic review and meta-analysis examined the safety of RYR extract supplementation [14]. The meta-analysis comprised 53 randomized clinical trials (*n* = 8535 participants) and primarily assessed the incidence of musculoskeletal disorders, whereas secondary outcomes included non-musculoskeletal symptoms and serious life events. In agreement with the findings of the present review, the use of RYR extract was not associated with an increased risk of musculoskeletal disorders (odds ratio [OR]: 0.94; 95% CI: 0.53–1.65). In contrast, RYR extract consumption was associated with a reduced risk of musculoskeletal side effects and serious complications compared to the control group. Therefore, RYR extract may be considered an effective and safe food drug at the recommended dosages that are available for the treatment of hypercholesterolemia. To date, the general recommendation is that RYR extract preparations should contain the component monacolin K in a dose of 3–10 mg for the treatment of hypercholesterolemia (EFSA Panel on Dietetic Products, Nutrition and Allergies) [39]. However, risks are not excluded even from this dosage, since not all preparations on the market that contain RYR extract contain the same ingredients.

Lastly, a concerning issue regarding preparations containing RYR extract is the cost of their preparation, as in some countries, RYR extract supplements may cost more than common statins [40]. Thus, the cost of RYR extract may have inhibitory effects on the wider use of this food drug. Given the current lack of cost-effectiveness studies on formulations containing RYR extract, future research is warranted to better assess whether the use of RYR extract can be adopted as a recommendation by major dyslipidemia prevention organizations and as a common practice by health promotion institutions and health policies.

### 4.3. Strengths and Limitations

Among the strengths of the present study is that it focused strictly on clinical trials which involved both intervention and control groups. Moreover, the included studies were on humans; thus, better conclusions can be drawn about the efficacy and safety of RYR extract when consumed by humans. Contrary to other meta-analyses, almost all eligible studies were double-blinded studies, thus lying in the highest part of the hierarchy of evidence pyramid. Moreover, we included more recently published studies in this field (*n* = 4 studies from 2019 up to 2021). Additionally, all eligible studies were of adequate quality (>10 points).

The main methodological limitation of the present study is the heterogeneity of the data with regard to the different RYR extract-containing formulations used in the intervention groups, the different dosage per study, the different total time of intervention and the variable follow-up periods. However, between-study heterogeneity was marginally non-statistically significant in all meta-analyses. Moreover, we applied strict inclusion and exclusion criteria which resulted in the exclusion of more than one hundred randomized controlled trials that tested the efficacy and safety of RYR. The different geographical origin of the participants should also be taken into account in the interpretation of the findings, as it may reflect different genetic profiles of the participants and it may subsequently impact drug metabolism. In addition, with the exception of total cholesterol and LDL-C, no meta-analyses were feasible on other indicators of hypercholesterolemia due to the limited number of publications (*n* = 1 to 3 studies). In the same context, no subgroup analyses were feasible at this stage to assess potential differences in effects due to variables such as age, sex, baseline cholesterol levels or dosage of red yeast rice extract. Due to the limited number of eligible studies, no generalizability of the results to broader populations or specific subpopulations is currently feasible, limiting the applicability of the findings. Lastly, the relatively small number of eligible studies did not allow us to assess issues of publication bias in the present study.

### 4.4. Future Research

To enrich the findings of the present study, future clinical trials are encouraged to investigate the association between RYR extract consumption and the levels of liver enzymes in order to assess any potential drug-induced hepatotoxicity. Further research into the dosage and ingredients of the formulations containing RYR extract is also suggested, so that the clinical benefits are maximized and the risks are minimized. The potential association of other ingredients added to RYR extract formulations with gastrointestinal symptoms also needs to be clarified more specifically, since side effects were also noted in the control groups in the present review. Finally, studies examining the potential administration of RYR extract preventively in cases of family history of hypercholesterolemia, as well as cost-effectiveness studies regarding the consumption of RYR extract, would be useful.

## 5. Conclusions

The consumption of RYR extract by people with hypercholesterolemia was associated with statistically significant reductions in total cholesterol and low-density lipoprotein cholesterol without an increased risk of life-threatening side effects. Further research on specific subpopulations and outcomes could establish a consensus on determining the clinical benefits of RYR extract consumption.

## Figures and Tables

**Table 1:** Descriptive characteristics of eligible studies.

Author, Year	Country	Sample Size (N)	Age Range in Years (Mean Age)	Mean BMI (kg/m^2^)
Feuerstein and Bjerke (2012) [20]	USA	18	>18 (57)	25
Minamizuka et al. (2021) [21]	Japan	18	20–80 (57.8)	24.4
Magno et al. (2018) [22]	Italy	20	18–75 (54.7)	30.3
Cicero et al. (2013) [23]	Italy	25	18–70 (51.2)	26.8
Ogier et al. (2013) [25]	France	39	18–55 (48)	23.7
Cicero et al. (2017) [24]	Italy	40	25–75 (52.2)	26.7
Domenech et al. (2019) [26]	Spain	40	35–75 (61.4)	27.1
Barrat et al. (2013b) [31]	France	45	18–65 (51.1)	23.9
Nafrialdi et al. (2019) [29]	Indonesia	49	18–60 (44.7)	27.5
Wang et al. (2019) [28]	Taiwan	50	25–70 (43.8)	25.1
Verhoeven et al. (2013) [30]	Belgium	54	>18 (55)	27.2
Mazza et al. (2015) [32]	Italy	66	18–70 (56)	27.4
Barrat et al. (2013a) [27]	France	99	18–65 (47.2)	23.4
Heinz et al. (2016) [33]	Germany	142	18–70 (57.3)	26.4

**Table 2:** Intervention characteristics of eligible studies.

Study	Weeks	Study Groups	Dose of Intervention	Statistical Analysis
Barrat et al. (2013b) [31]	4	One intervention and one control	167 mg RYR (0.67 mg monacolin K)	Two-tailed *t*-test and ANCOVA
Nafrialdi et al. (2019) [29]	4	One intervention and one control	10 mg monacolin K	Paired-*t* or Wilcoxon test, unpaired-*t* or Mann–Whitney test
Feuerstein and Bjerke (2012) [20]	6	One intervention	1200 mg RYR	Paired *t* tests, Kolmogorov–Smirnov and Shapiro–Wilk
Verhoeven et al. (2013) [30]	8	One intervention and one control	5.025 mg monacolin K	Paired *t*-test, chi^2^, Fischer’s test or Mann–Whitney U
Domenech et al. (2019) [26]	12	One intervention and one control	10 mg monacolin K	Kolmogorov–Smirnov, chi-square, ANOVA, or Mann–Whitney U, Wilcoxon
Heinz et al. (2016) [33]	12	One intervention and one control	200 mg RYR (3 mg monacolin K)	Kolmogorov–Smirnov, *t* test, Mann–Whitney U, Wilcoxon, χ^2^
Minamizuka et al. (2021) [21]	12	One intervention and one control	200 mg RYR daily (2 mg monacolin K)	*t* test, Wilcoxon, and Pearson’s correlation
Barrat et al. (2013a) [27]	16	One intervention and one control	167 mg RYR (0.4% monacolin K)	Two-tailed *t*-test and ANCOVA
Cicero et al. (2013) [23]	16	One intervention and one control	10 mg monacolins by *Monascus purpureus*	Student *t*-test, Fisher exact, χ^2^ and ANOVA
Ogier et al. (2013) [25]	16	One intervention and one control	166.67 mg RYR (0.4% monacolin K)	Student *t*-test, χ^2^ test and Fisher exact test
Cicero et al. (2017) [24]	18	One intervention and one control	200 mg RYR (10 mg monacolin K)	Student *t*-test and Mann–Whitney U test
Wang et al. (2019) [28]	18	Two interventions and one control	RYR fortified with monacolin K (8 mg): daily dose of 400 mg or RYR fortified with GABA: daily dose of 335 mg (GABA 0.14 mg)	Kruskal–Wallis, chi-square and ANOVA Friedman
Magno et al. (2018) [22]	20	One intervention and one control	One tablet daily including monacolin K, L-arginin, Q10 and ascorbic acid (treatment A), and one tablet daily with L-arginin, Q10 and ascorbic acid (treatment N)	Paired *t* test and ANOVA
Mazza et al. (2015) [32]	24	One intervention and one control	RYR (3 mg monacolin K)	ANCOVA and χ^2^ test

Abbreviations: RYR: red yeast rice; ANCOVA: analysis of covariance; ANOVA: analysis of variance; GABA: gamma-aminobutyric acid.

**Table 3:** The results on the association between red yeast rice extract consumption and parameters of the lipid profile.

Results					
Study	TC	LDL-C	HDL-C	TGs	Other Parameters
Barrat et al. (2013a) [27]	NR	−22 mg/dL (−31.0 to −12.0)	NS	NR	apoA-I: +0.04 g/L (0.00, 0.09 g/L; *p* = 0.0347)
Cicero et al. (2013) [23]	−12.45%, (−16.19, −8.71)	−21.99% (CI: −26.63, −17.36)	NS	NR	Non-HDL-c: −14.67% (−19.22, −10.11) MMP-2: −28.05% (−35.18, −20.93) MMP-9: −27.19% (−36.21, −18.15) hs-CRP: −23.77% (−30.54, −17.01)
Cicero et al. (2017) [24]	−34.1 mg/dL (−56.8; −11.3 mg/dL; *p* < 0.001)	−30.3 mg/dL (−49.7; −7.4; *p* < 0.001)	NS	NR	Non-HDL: −31.2 mg/dL (−45.5, −8.1; *p* < 0.001) SGOT: −2.3 U/L (−5.7, −0.9; *p* < 0.024) SGPT: −6.8 U/L (−10.4, −1.4; *p* < 0.011) hs-CRP: −0.4 (−0.9; −0.1, *p* < 0.019)
Domenech et al. (2019) [26]	−14.1%	−19.7%	NS	NR	NR
Feuerstein and Bjerke (2012) [20]	−46 mg/dL, *p* < 0.05	−53 mg/dL, *p* < 0.05	NS	NR	NR
Heinz et al. (2016) [33]	−11.2%	−14.1%, *p*< 0.001	NR	−5.0%	NR
Mazza et al. (2015) [32]	−19.2%, *p* < 0.001	−17.4%, *p* < 0.001	NS	TGs: −16.3%, *p* < 0.001	NR
Minamizuka et al. (2021) [21]	0.00 ± 0.75 mmol/L vs. −0.92 ± 0.57 mmol/L, *p* = 0.014	−0.20 [−0.64, 1.19] mmol/L vs. −0.96 [−1.05, −0.34] mmol/L, *p* = 0.030	NR	NR	non-HDL-c: 0.28 (−0.66, 0.56) mmol/L vs. −0.98 (−1.16, −0.82) mmol/L, *p* = 0.023 ApoB: 0.03 ± 0.16 g/L vs. −0.18 ± 0.11 g/L, *p* = 0.011
Nafrialdi et al. (2019) [29]	Reduction from 214.5 to 186.5 mg/dl	Reduction from 149.6 to 122.5 mg/dL	NR	NR	NR
Ogier et al. (2013) [25]	−14.1% (95% CI: −10.1 to −18.0%; *p* < 0.001)	−21.4% (95% CI: −13.3 to −24.9%; *p* < 0.001)	NS	−12.2% (95% CI: −24.4, −0.1%; *p* < 0.05)	NR
Verhoeven et al. (2013) [30]	NR	−22.17%	NR	NR	NR
Barrat et al. (2013b) [31]	6-TAB: −0.23 g/L; 95% CI: −0.42 to −0.04 g/L; *p* = 0.0425. 3-TAB: −0.27 g/L; 95% CI: −0.46 to −0.08 g/L; *p* = 0.0176	6-TAB: −0.21 g/L; 95% CI: −0.38 to −0.03 g/L; *p* = 0.0217 3-TAB: −0.25 g/L; 95% CI: −0.42 to −0.07 g/L; *p* = 0.0071	NS	NS	NR
Magno et al. (2018) [22]	A: −42.8 mg/dL Ν: −34.1 mg/dL *p* < 0.0001	A: −25.6% Ν: −23.3%	NS	−17.3 mg/dL, *p* < 0.05, in intervention group (A)	NR
Wang et al. (2019) [28]	Reduction in monacolin K from 237 (203–285) to 192.5 (149–220)	Reduction in monacolin K group from 153 (111–206) to 122 (71–138)	NS	NS	NR

Abbreviations: Apo: apolipoprotein; HDL-C: high-density lipoprotein cholesterol; hs-CRP: high-sensitivity C-reactive protein; LDL-C: low-density lipoprotein cholesterol; MMP: matrix metalloproteinase; NS: non-statistically significant association; NR: not reported; SGOT: glutamic-oxaloacetic transaminase; SGPT: glutamic pyruvic transaminase; TC: total cholesterol; TGs: triglycerides.

**Table 4:** Reported adverse effects in intervention groups (only red yeast rice extract consumption) and in both intervention and control groups.

Study	Only Intervention Group	Both Intervention and Control Groups
Minamizuka et al. (2021) [21]	-	Rare: polyuria, abdominal pain, drowsiness, itching, nausea, constipation and numbness of the fingertips
Nafrialdi et al. (2019) [29]	-	Rare: myalgia, weakness, cramps, insomnia, erectile dysfunction, pruritus and abdominal pain
Barrat et al. (2013a) [27]	Very rare: abdominal pain and bitter taste	-
Barrat et al. (2013b) [31]	Very rare: cystitis, bloating, nausea and headache	-
Heinz et al. (2016) [33]	Rare: flatulence Very rare: diarrhea, constipation and feeling of gastric fullness	Rare: flatulence Very rare: diarrhea, constipation and feeling of gastric fullness
Magno et al. (2018) [22]	Rare: constipation, flatulence and diarrhea Very rare: eczema, headache, myalgia and increased CK levels	-
Mazza et al. (2015) [32]	Very rare: increased CK levels and dyspepsia	-
Wang et al. (2019) [28]	Rare: increased SGOT/SGPT ratio	-

## Data Availability

The original contributions presented in the study are included in the article and Supplementary Materials; further inquiries can be directed to the corresponding author.

## Supplementary Materials

The following supporting information can be downloaded at: https://www.mdpi.com/article/10.3390/nu16101453/s1, Table S1: Preferred Reporting Items for Systematic Reviews and Meta-analysis (PRISMA) 2020 item checklist; Table S2: Study quality assessment.

## Appendix A. Search Algorithm

(Hypercholesterolemia OR High Cholesterol OR High-cholesterol OR High Lipoprotein OR Low-density Lipoprotein OR LDL) AND (Red yeast Rice OR Red-yeast-Rice OR R-Y-R OR RYR OR Red Yeast Rice extract) AND (safety OR Side effects OR Adverse effects OR Adverse events) AND (Efficacy OR Effectiveness OR Advantages OR Benefit) AND (Clinical Study OR Clinical Trial OR Randomised Controlled Trial).
