# Supplementary material for: Safety and Efficacy of the Consumption of the Nutraceutical “Red Yeast Rice Extract” for the Reduction of Hypercholesterolemia in Humans: A Systematic Review and Meta-Analysis
Source: Nutrients. 2024 May 11;16(10):1453. doi: 10.3390/nu16101453 (PMC11124448; doi:10.3390/nu16101453)
Supplement: Supplementary file 1 [file nutrients-16-01453-s001.zip › nutrients-2984373-supplementary.pdf]

Supplementary Table S1. Preferred Reporting Items for Systematic Reviews and Meta-analysis (PRISMA) 2020 item checklist.

Section and topic	Item #	Checklist item	Location where item is reported
**Title**			
Title	1	Identify the report as a systematic review.	Line 4
**Abstract**			
Abstract	2	See the PRISMA 2020 for Abstracts checklist	Lines 10-25
**Introduction**			
Rationale	3	Describe the rationale for the review in the context of existing knowledge.	Lines 29-53
Objectives	4	Provide an explicit statement of the objective(s) or question(s) the review addresses.	Lines 54-60
**Methods**			
Eligibility criteria	5	Specify the inclusion and exclusion criteria for the review and how studies were grouped for the syntheses.	Lines 76-82
Information sources	6	Specify all databases, registers, websites, organisations, reference lists and other sources searched or consulted to identify studies. Specify the date when each source was last searched or consulted.	Lines 62-73
Search strategy	7	Present the full search strategies for all databases, registers and websites, including any filters and limits used.	Lines 73-74
Selection process	8	Specify the methods used to decide whether a study met the inclusion criteria of the review, including how many reviewers screened each record and each report retrieved, whether they worked independently, and if applicable, details of automation tools used in the process.	Lines 67-73
Data collection process	9	Specify the methods used to collect data from reports, including how many reviewers collected data from each report, whether they worked independently, any processes for obtaining or confirming data from study investigators, and if applicable, details of automation tools used in the process.	Lines 84-87
Data items	10a	List and define all outcomes for which data were sought. Specify whether all results that were compatible with each outcome domain in each study were sought (e.g. for all measures, time points, analyses), and if not, the methods used to decide which results to collect.	Lines 84-87
	10b	List and define all other variables for which data were sought (e.g. participant and intervention characteristics, funding sources). Describe any assumptions made about any missing or unclear information.	Lines 84-87

Section and topic	Item #	Checklist item	Location where item is reported
Study risk of bias assessment	11	Specify the methods used to assess risk of bias in the included studies, including details of the tool(s) used, how many reviewers assessed each study and whether they worked independently, and if applicable, details of automation tools used in the process.	Lines 88-100
Effect measures	12	Specify for each outcome the effect measure(s) (e.g. risk ratio, mean difference) used in the synthesis or presentation of results.	Lines 84-87
Synthesis methods	13a	Describe the processes used to decide which studies were eligible for each synthesis (e.g. tabulating the study intervention characteristics and comparing against the planned groups for each synthesis (item #5)).	Lines 102-108
	13b	Describe any methods required to prepare the data for presentation or synthesis, such as handling of missing summary statistics, or data conversions.	Lines 109-115
	13c	Describe any methods used to tabulate or visually display results of individual studies and syntheses.	Lines 109-115
	13d	Describe any methods used to synthesise results and provide a rationale for the choice(s). If meta-analysis was performed, describe the model(s), method(s) to identify the presence and extent of statistical heterogeneity, and software package(s) used.	Lines 109-115
	13e	Describe any methods used to explore possible causes of heterogeneity among study results (e.g. subgroup analysis, meta-regression).	Lines 109-115
	13f	Describe any sensitivity analyses conducted to assess robustness of the synthesised results.	Lines 109-115
Reporting bias assessment	14	Describe any methods used to assess risk of bias due to missing results in a synthesis (arising from reporting biases).	Lines 109-115
Certainty assessment	15	Describe any methods used to assess certainty (or confidence) in the body of evidence for an outcome.	Lines 109-115
**Results**			
Study selection	16a	Describe the results of the search and selection process, from the number of records identified in the search to the number of studies included in the review, ideally using a flow diagram (see Fig. 1).	Lines 119-129
	16b	Cite studies that might appear to meet the inclusion criteria, but which were excluded, and explain why they were excluded.	Lines 119-129
Study characteristics	17	Cite each included study and present its characteristics.	Lines 119-129

Section and topic	Item #	Checklist item	Location where item is reported
Risk of bias in studies	18	Present assessments of risk of bias for each included study.	Lines 127-129
Results of individual studies	19	For all outcomes, present, for each study: (a) summary statistics for each group (where appropriate) and (b) an effect estimate and its precision (e.g. confidence/credible interval), ideally using structured tables or plots.	Lines 130-142
Results of syntheses	20a	For each synthesis, briefly summarise the characteristics and risk of bias among contributing studies.	Lines 144-159
	20b	Present results of all statistical syntheses conducted. If meta-analysis was done, present for each the summary estimate and its precision (e.g. confidence/credible interval) and measures of statistical heterogeneity. If comparing groups, describe the direction of the effect.	Lines 144-159
	20c	Present results of all investigations of possible causes of heterogeneity among study results.	Lines 144-159
	20d	Present results of all sensitivity analyses conducted to assess the robustness of the synthesised results.	Lines 160-211
Reporting biases	21	Present assessments of risk of bias due to missing results (arising from reporting biases) for each synthesis assessed.	Lines 144-159
Certainty of evidence	22	Present assessments of certainty (or confidence) in the body of evidence for each outcome assessed.	Lines 144-159
**Discussion**			
Discussion	23a	Provide a general interpretation of the results in the context of other evidence.	Lines 227-299
	23b	Discuss any limitations of the evidence included in the review.	Lines 300-317
	23c	Discuss any limitations of the review processes used.	Lines 300-317
	23d	Discuss implications of the results for practice, policy, and future research.	Lines 318-336
**Other information**			
Registration and protocol	24a	Provide registration information for the review, including register name and registration number, or state that the review was not registered.	Lines 62-65
	24b	Indicate where the review protocol can be accessed, or state that a protocol was not prepared.	Lines 62-65
	24c	Describe and explain any amendments to information provided at registration or in the protocol.	Lines 62-65
Support	25	Describe sources of financial or non-financial support for the review, and the role of the funders or sponsors in the review.	Page 9

Section and topic	Item #	Checklist item	Location where item is reported
Competing interests	26	Declare any competing interests of review authors.	Page 9
Availability of data, code, and other materials	27	Report which of the following are publicly available and where they can be found: template data collection forms; data extracted from included studies; data used for all analyses; analytic code; any other materials used in the review.	Page 9

Supplementary Table S2. Study Quality Assessment

Quality Criteria	Study-quality assessment													
	Barrat et al. (2013a)	Barrat et al. (2013b)	Cicero et al. (2013)	Cicero et al. (2017)	Domenech et al. (2019)	Feuerstein & Bjerke (2012)	Heinz et al. (2016)	Magno et al. (2018)	Mazza et al. (2015)	Minamizuka et al. (2021)	Nafrialdi et al. (2019)	Ogier et al. (2013)	Verhoeven et al. (2013)	Wang et al. (2019)
Description as RCT	1	1	1	1	1	1	1	1	1	1	1	1	1	1
Adequate use of RCT methodology	1	1	1	1	1	1	1	1	0	1	1	1	1	1
Treatment allocation concealed	1	1	1	1	1	1	1	1	1	1	1	1	1	1
Participants and providers blinded to treatment group assignment	1	1	1	1	1	0	1	0	0	0	1	1	1	1
People assessing the outcomes blinded to the participants' group assignments	1	1	1	1	1	0	1	0	0	0	1	1	1	1
Groups similar at baseline on important characteristics that could affect outcomes	1	1	1	1	1	0	1	1	1	1	0	1	0	0
≤20% overall drop-out rate from the study at endpoint	1	1	1	1	1	1	1	1	1	1	1	1	1	1
<15% differential drop-out rate at endpoint	1	1	1	1	1	1	1	1	1	1	1	1	1	1
High adherence to the intervention protocols	1	1	1	1	1	1	1	1	1	1	1	1	1	1
Other interventions avoided or similar in the groups	1	1	1	1	1	1	1	1	1	1	1	1	1	1
Outcomes assessed using valid and reliable measures	1	1	1	1	1	1	1	1	1	1	1	1	1	1
Sample size power ≥ 80%	1	1	1	0	1	1	1	1	1	1	1	0	1	1
Outcomes reported or subgroups analyzed prespecified	1	1	1	1	1	1	1	1	1	1	1	1	0	1
Use of intention-to-treat analysis	1	1	1	1	1	1	1	1	0	1	1	1	0	0
Total score	14	14	14	13	14	11	14	12	10	12	13	13	11	12

RCT: randomized controlled trial
